# Citizens’ Attitudes to Contact Tracing Apps

**DOI:** 10.1017/XPS.2020.30

**Published:** 2020-09-02

**Authors:** Laszlo Horvath, Susan Banducci, Oliver James

**Affiliations:** Department of Politics, University of Exeter, Exeter, UK; Twitter: @_lhorvath, @femalebrain, @_Oliver_James_

**Keywords:** Digital contact tracing, privacy, data breach, conjoint experiment

## Abstract

Citizens’ concerns about data privacy and data security breaches may reduce the adoption of COVID-19 contact tracing mobile phone applications, making them less effective. We implement a choice experiment (conjoint experiment) where participants indicate which version of two contact tracing apps they would install, varying the apps’ privacy-preserving attributes. Citizens do not always prioritise privacy and prefer a centralised National Health Service system over a decentralised system. In a further study asking about participants’ preference for digital-only vs human-only contact tracing, we find a mixture of digital and human contact tracing is supported. We randomly allocated a subset of participants in each study to receive a stimulus priming data breach as a concern, before asking about contact tracing. The salient threat of unauthorised access or data theft does not significantly alter preferences in either study. We suggest COVID-19 and trust in a national public health service system mitigate respondents’ concerns about privacy.

## Introduction

Contact tracing mobile applications can help slow the spread of COVID-19 (Ferretti et al. 2020). However, citizens’ concerns about data privacy and data security breaches may reduce adoption below the required coverage to be effective (Ada Lovelace Institute 2020; Liu and Carter 2018). We analyse the determinants of citizens’ attitudes to these contact tracing apps. In *Study 1*, we implement a choice experiment (conjoint experiment) where participants indicated which version of two contact tracing apps they would be most likely to install. We vary the privacy-preserving attributes of the apps and estimate their effects on adoption. In *Study 2*, participants indicate a preference for digital-only vs human-only contact tracing. To assess the salience of data breaches as an issue for adoption, we randomly allocated a subset of participants in each study to receive a stimulus priming data breach as a concern, before asking about contact tracing.

Under current pandemic conditions, we find that citizens do not always prioritise privacy but give high preference to a centralised system led by the National Health Service (NHS) over a decentralised system (see also Wiertz et al. 2020). Citizens tend to support a mixture of contact tracing done digitally with limited human involvement. The salient threat of unauthorised access or data theft does not significantly alter either set of preferences.

**Figure 1:**
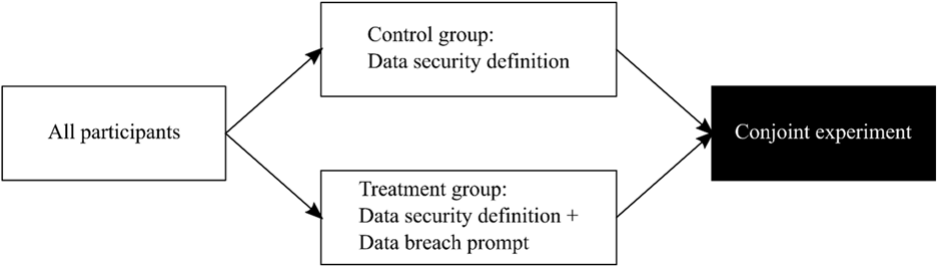
Overview of *Study 1*.

**Figure 2:**
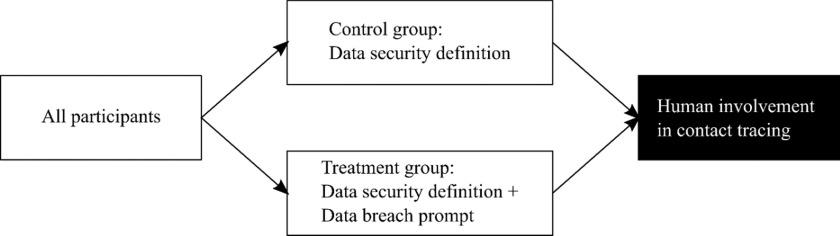
Overview of *Study 2*.

**Figure 3:**
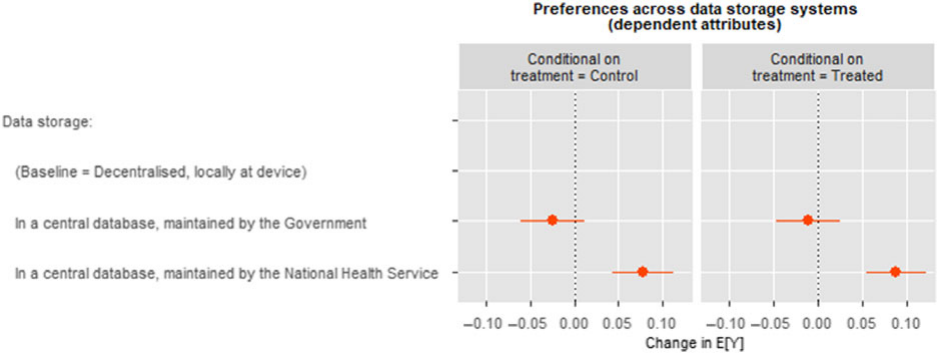
Treatment effects on preference of data storage systems. *Note:* Treatment is exposure to stimulus raising awareness of data breach. AMCE values calculated with the cjoint (Hainmueller et al. 2014) package in R. For ATE values (coefficients) see Table 2.

**Figure 4:**
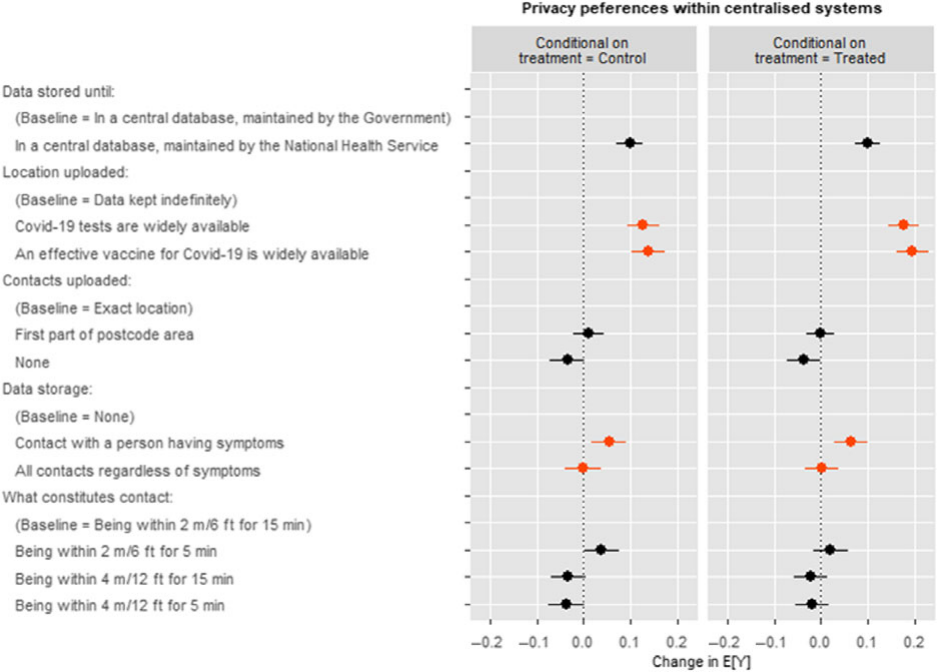
Treatment effects on privacy preferences within centralised systems. *Note:* Treatment is exposure to stimulus raising awareness of data breach. AMCE values calculated with the cjoint (Hainmueller et al. 2014) package in R. For ATE values (coefficients) see Table 3.

**Figure 5:**
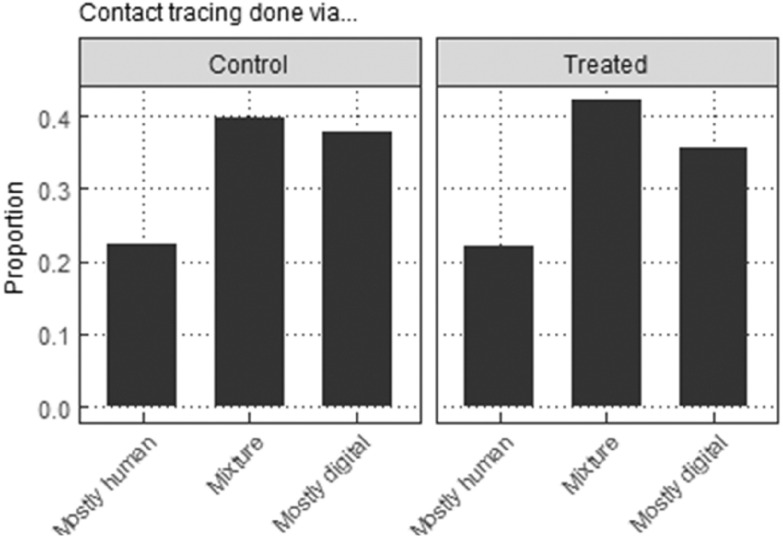
Preference of digital vs human contact tracing per treatment group. *Note*: Treatment is exposure to stimulus raising awareness of data breach.

## Theory and hypotheses

Research on the adoption of technology similar to mobile phone contact tracing applications has shown that users’ concern about data security and privacy can reduce acceptance. A study of predictors of individuals’ adoption of healthcare wearable devices found that individuals’ privacy perceptions were an important part of their calculations about the use of the technology (Li et al. 2016). This leads to our first hypothesis:

**Baseline preference of privacy.** We hypothesise a baseline preference of more privacy-preserving contact tracing applications.

The process of contact tracing using apps in practice supplements traditional human contact tracing. There is little direct evidence about this issue for COVID-19, but the broader literature on algorithm aversion suggests that people tend to prefer human involvement in systems even if they perform less well (Dietvorst, Simmons, and Massey 2015). We, therefore, propose the following hypothesis:

**Baseline preference of human contact tracing.** We hypothesise that citizens prefer more human involvement over digital-only contact tracing.

The concerns of users about privacy and preference for human contact tracing lead us to further examine whether making the possibility of data breaches more salient strengthens these baseline preferences, leading to a third hypothesis:

**Saliency of the data breach.** We hypothesise that preferences of privacy-preserving contact tracing, as well as human contact tracing, are strengthened for individuals who consider data breach as a realistic threat.

The international experience with COVID-19 has shown that citizens’ responses and willingness to engage with public health measures are affected by broader socio-political attitudes. Recently, evidence has emerged about differences based on partisanship (e.g. Utych 2020) and gender (Palmer and Peterson 2020). In the UK context, where our studies are based, there is less clear evidence about partisan divides but the issue of other political attitudes towards the public authorities proposing the use of technology is still salient. Previous studies have found that trust in organisations is a factor influencing the intention to use related digital government technologies (van Velsen et al. 2015). For these reasons, we include measures of trust in the NHS, and trust in the UK government’s handling of COVID-19. In each case, higher trust is expected to increase acceptance of privacy reducing and more technology-reliant aspects of the mobile phone app.

Globally, digital contact tracing is being rolled out with a variety of system architectures that have different implications for privacy and data security. The core functionalities of a *centralised system* are performed by a central server processing user data, which is managed by a health authority and can, subject to permissions, notify an infected user’s contacts of exposure (Ahmed et al. 2020, 134578–134580; Martin et al. 2020). A *decentralised system*, on the other hand, has most of its core functionalities performed by users’ devices including exposure notifications (Ahmed et al. 2020, 134580–134581). The privacy implications of these two systems have often been discussed as a trade-off with other attributes (see also Cioroianu and Dal 2020, for an overview). While decentralised systems are recommended for having more overall privacy-preserving features than centralised systems,^1^ the lack of central oversight does limit human involvement in the process of contact tracing. This might be problematic while contact tracing apps tend to perform with poor accuracy (Briers 2020). In contrast, whereas centralised systems do have the ability to integrate digital with human contact tracing and research (by design, but in practice may be a legislative feature), their data servers are vulnerable to the data breach that involves more sensitive protected data.

## Methods

### Subjects and context^2^

*Study 1* uses an online panel of *N* = 1,504 from Dynata, targeting a diversity of respondents representative of the UK as of its 2011 census^3^; *Study 2* uses a smaller, *N* = 809 panel from Prolific Academic, with similar sample demographics,^4^^,^ ^5^ Data collection occurred on 18 May 2020 to 23 May 2020. During this period, the UK had no official (government-backed) contact tracing app available for public use, except for a trial version released on the Isle of Wight exclusively. That application was one of the centralised systems as outlined above. The UK’s next contact tracing app to enter a new trial phase will be built on a decentralised system (Department of Health & Social Care 2020).

### Dependent measures

In *Study 1 conjoint experiment,* respondents were asked to choose one of two COVID-19 contact tracing apps to install, with their data privacy and security attributes varying. Each respondent made a series of five such selections.

In *Study 2*, the dependent measure is the respondents’ preferred amount of human involvement in the process of COVID-19 contact tracing. This is a rating scale ranging from human-only contact tracing (1) to digital-only contact tracing (7).

### Treatment: Data breach stimulus

Both groups in each study received a brief text about data security including its definition as “a set of standards and technologies that protect data from intentional or accidental destruction, modification or disclosure.” The treatment group additionally got a text about data breaches becoming “more common,” giving examples: “theft of personal data, devices containing personal data being lost or stolen.”^6^ Figures 1 and 2 show the placement of treatment stimuli in the two studies, respectively.

### Conjoint experiment

The conjoint experiment enabled us to assess the causal impact of multiple attributes related to privacy and data security: *data storage until when data are stored, what kinds of contacts and what specificity of location is uploaded and what constitutes contact*.

Conventional conjoint experiments randomise and display all attributes independently. Our challenge, however, was to capture two vastly different implementations of digital contact tracing with their privacy options fundamentally incompatible with each other, restricting the option of independent randomisation. Decentralised systems of digital contact tracing use minimal data sharing across devices whereas centralised systems use data sharing between devices and a data server. As explained above, decentralised systems may preserve privacy better but are not integrated with human contact tracing and research in contrast to centralised systems that can be integrated but may consequently be seen as vulnerable to data breaches.

We address the issue using *dependent attributes*. Respondents evaluate five pairs of potential contact tracing applications that compare either a decentralised system that simply does not store contact or location data with a centralised system that stores at least one of these, or two centralised systems with varying privacy attributes excluding the possibility of no data storage. In this way, attributes presented in Table 1 below are heavily system-dependent.

**Table 1:** Privacy attributes depending on data storage system (dependent attributes)

Attributes and attribute levels			
**Data storage**			
Decentralised, locally on device	In a central database: NHS		In a central database: Gov’t
**Purpose of app (explanatory attribute only)**			
Notify user directly of exposure	Inform human contact tracer to examine user’s exposure to virus		
**Data stored until**			
Not stored	Indefinitely	Tests widely available	Vaccine available
**Location uploaded**			
None	Exact location		1st part of postcode area
**Contacts uploaded**			
None	All contacts		Person with symptoms
**What constitutes a contact**			
6ft/5 min	6ft/15 min	12ft/5 min	12ft/15 min

*Notes*: A third of all binary comparisons were between a centralised system (privacy attributes varying) and a decentralised system (privacy attributes not varying), and two-thirds between two centralised systems (privacy attributes varying, greyed cells). Privacy-varying attributes reported on latter subsample.

**Table 2:** Data storage models

	Treatment	Moderator 1: NHS trust	Moderator 2: Gov’t performance
*Dependent variable: Pr(profile chosen)*			
Intercept (baseline: decentralised, stored on device)	−0.08	0.31*	0.18*
	(0.05)	(0.15)	(0.09)
Covariate (see notes)	−0.04	−0.05**	−0.11**
	(0.06)	(0.02)	(0.03)
In a central database, maintained by the government	−0.10	−0.35	−0.72***
	(0.06)	(0.20)	(0.12)
In a central database, maintained by the NHS	0.31***	−0.49*	0.24
	(0.06)	(0.20)	(0.12)
Covariate × in a central database, maintained by the government	0.05	0.03	0.25***
	(0.08)	(0.02)	(0.04)
Covariate × in a central database, maintained by the NHS	0.04	0.10***	0.04
	(0.08)	(0.02)	(0.04)
AIC	20,737.13	20,718.81	20,356.22
Log likelihood	−10,362.57	−10,353.41	−10,172.11
Num. obs.	15,040	15,040	14,790

***p < 0.001, **p < 0.01, *p < 0.05.

Pooled GLM estimates with standard errors clustered on respondent level.

*Note*: For simplified display, “Covariate” means “Treatment” in the first,

“Trust in NHS” in the second and “Gov’t performance” in the third column.

**Table 3:** Treatment and moderator effects on preference within centralised systems

	Treatment	Moderator 1: NHS trust	Moderator 2: Gov’t performance
*Dependent variable: Pr(profile chosen)*			
Intercept	−0.57***	−0.82*	−1.21***
	(0.09)	(0.32)	(0.20)
Covariate (see notes)	−0.15	0.02	0.22**
	(0.13)	(0.04)	(0.07)
In a central database, maintained by the NHS	0.41***	−0.12	0.98***
	(0.06)	(0.19)	(0.12)
Covid-19 tests are widely available	0.52***	0.16	0.79***
	(0.07)	(0.23)	(0.14)
An effective vaccine for Covid-19 is widely available	0.56***	0.46	0.96***
	(0.07)	(0.23)	(0.15)
First part of postcode area	0.04	0.27	−0.10
	(0.06)	(0.22)	(0.14)
None	−0.14	0.68**	−0.07
	(0.08)	(0.26)	(0.16)
Contact with person having symptoms	0.23**	0.44	0.31*
	(0.08)	(0.25)	(0.16)
All contacts regardless of symptoms	−0.00	−0.08	0.01
	(0.08)	(0.26)	(0.16)
Within 2 m/6 feet for 5 min	0.16*	0.26	0.29
	(0.08)	(0.27)	(0.17)
Within 4 m/12 feet for 15 min	−0.13	0.14	−0.14
	(0.08)	(0.26)	(0.17)
Within 4 m/12 feet for 5 min	−0.15	0.23	−0.13
	(0.08)	(0.27)	(0.17)
Covariate × in a central database, maintained by the NHS	0.01	0.06**	−0.22***
	(0.08)	(0.02)	(0.04)
Covariate × Covid-19 tests are widely available	0.21*	0.06*	−0.06
	(0.10)	(0.03)	(0.05)
Covariate × an effective vaccine for Covid-19 is widely available	0.24*	0.03	−0.10
	(0.10)	(0.03)	(0.05)
Covariate × first part of postcode area	−0.05	−0.03	0.04
	(0.09)	(0.03)	(0.05)
Covariate × none	−0.01	−0.10**	−0.03
	(0.11)	(0.03)	(0.06)
Covariate × contact with person having symptoms	0.04	−0.02	−0.02
	(0.11)	(0.03)	(0.06)
Covariate × all contacts regardless of symptoms	0.01	0.01	−0.01
	(0.11)	(0.03)	(0.06)
Covariate × within 2 m/6 feet for 5 min	−0.07	−0.02	−0.07
	(0.11)	(0.03)	(0.06)
Covariate × within 4 m/12 feet for 15 min	0.04	−0.03	0.01
	(0.11)	(0.03)	(0.06)
Covariate × within 4 m/12 feet for 5 min	0.07	−0.04	0.00
	(0.11)	(0.03)	(0.06)
AIC	14,776.57	14,756.40	14,494.44
Log likelihood	−7,366.29	−7,356.20	−7,225.22
Num. obs.	10,950	10,950	10,766

***p < 0.001, **p < 0.01, *p < 0.05.

Pooled GLM estimates with standard errors clustered on respondent level.

*Note*: For simplified display, “Covariate” means “Treatment” in the first,

“Trust in NHS” in the second and “Gov’t performance” in the third column.

**Table 4:** Treatment effects on preferred amount of human involvement in contact tracing

	Pr(*y* = Mostly human)	Pr(*y* = Mixture)	Pr(*y* = Mostly digital)
Intercept	−1.24*	−0.42*	−0.50*
	(0.12)	(0.10)	(0.10)
Treated	−0.01	0.10	−0.09
	(0.17)	(0.14)	(0.15)
AIC	864.11	1,098.15	1,067.26
Log likelihood	−430.05	−547.07	−531.63
Num. obs.	809	809	809

*p < 0.001.

As attributes are not fully independent we (1) present a comparison of effect sizes on the data storage attribute alone, independently from the privacy attributes, and then (2) present the effect of the rest of the privacy attributes separately on respondents who compared two centralised systems.^7^

### Moderators

We include the following as moderators of conjoint preferences: trust in the NHS and satisfaction with the government’s handling of coronavirus.^8^

## Results

### *Study 1*

#### Descriptive results

Across all attributes, respondents do not systematically prefer more privacy. For data storage, the NHS led centralised system is preferred in 55.94% of binary comparisons compared with the centralised system led by the UK government (45.85%) and the decentralised system (47.63%) despite the NHS system being potentially displayed with attributes more intrusive to privacy.^9^

#### Treatment effects: Data storage

In the pooled model across all conjoint choices (five tasks, two profiles per task displayed by respondents thus *N* = 15,040) with standard errors clustered on the respondent level, we found no difference between a preference for data storage and exposure to the data breach stimulus, see Figure 3.

#### Treatment effects within centralised data storage systems

An *N* = 10,950 app profiles described a centralised system with further attributes relating to privacy varying. In this subset of the data, our model finds no treatment effects relating to exposure to the data breach stimulus except some evidence that the stimulus may have further strengthened respondents’ preference to store data until vaccines or tests are available over indefinite data storage, see Figure 4.

#### Trust in NHS as a moderator

In two similar pooled models as above, across data storage systems as well as within centralised systems we find that although high trust in the NHS strengthens preferences for an NHS-led centralised system, low trust in the NHS does not mean clear support for a decentralised system (or a centralised one maintained by the government). Within centralised systems, trust in the NHS motivates respondents to give up more privacy.

#### Government performance as moderator

Satisfaction with the government’s performance in handling COVID-19 moderated preferences given to a centralised system maintained by the UK government. Across the spectrum, however, the NHS-led centralised system remains the clear preference in the majority of comparisons.

### *Study 2*

*Study 2* repeated the data breach stimulus asking respondents about their preferred amount of human involvement in the process of contact tracing. The majority of citizens prefer a mixture between human-led and digital, with greater proportions preferring “Mostly digital” to “Mostly human.”^10^ We find no significant treatment effects for exposure to data breaches, see Figure 5 and Table 4.

## Discussion and Conclusions

Citizens prefer a balanced (human plus digital) approach to contract tracing. Privacy concerns were not as influential on the choice of the digital app as initially expected and as indicated by past research. Privacy concerns were overridden by trust in the NHS and the NHS centralised app is preferred to both the centralised government app and the decentralised system. The NHS has strong support amongst the UK public; support and research on other public services have found users have greater willingness to cooperate in the co-production of public services delivered by public organisations when compared with services delivered under contract to private companies (James and Jilke 2020). Our findings are consistent with this line of research and demonstrate that when a trusted public health provider is involved in the development and deployment of the tracing app it can bring about the cooperation of the public necessary for its successful use in reducing the spread of infection.

Our results suggest two considerations for future research. First, to further understand the role of health care providers, research should examine the effect of institutional differences on coproduction, including whether the organisation is public or privately owned. The unique status of the NHS with its current high regard among the British public may not translate to coproduction in all other jurisdictions. Secondly, variation in the perceptions of the salience of the COVID-19 threat across different countries and populations might explain how the public responds to privacy concerns. The data breach treatment does not influence outcomes, possibly because of crisis perceptions which were likely high in the initial phase of the pandemic. Potential changes in responses over time as the pandemic develops and differences in findings between jurisdictions with different public health systems are particularly important topics for future research.

## Footnotes

1 A central data server is especially vulnerable to a single point of failure (Ahmed et al. 2020, 134585) but as Baumgärtner et al. (2020) show, decentralised systems are vulnerable to potential profiling of individual user locations.

2 The research design presented here was reviewed and approved by the University of Exeter College of Social Sciences and International Studies Ethics Committee, and pre-registered at Aspredicted.org Study No. #41234 prior to data collection.

3 Our target sample size for *Study 1* was 1,500 to allow us for a minimum detectable effect size of approximately 5% on a four-level attribute across five discrete choice tasks.

4
*Study 2* requires substantially fewer observations based on power considerations only. We targeted 800 towards the lower end *N* where the data provider offered the option of representative demographics.

5 Distribution of age, gender and region of residence are summarised in supplementary material. Other than compliance with our demographic quotas (managed by the data supplier) and indication of informed consent, there were no additional inclusion criteria to participate and paid respondents self-selected to participate.

6 We examine compliance with treatment in supplementary material.

7 We include more explanation in supplementary material.

8 “Please tell me on a scale of 0-10 how much you personally trust the National Health Service [NHS] where 0 means you do not trust the NHS at all, and 10 means you have complete trust,” and “How well or badly do you think the UK government is handling the coronavirus (Covid-19) outbreak?” expressed on a 1 (Very badly) to 4 (Very well) scale.

9 We show the obtained distribution of all conjoint preferences in supplementary material.

10 We show the original distribution of preferences descriptively in supplementary material.

## Supplementary material

For supplementary material accompanying this paper visit https://doi.org/10.1017/XPS.2020.30. click here to view supplementary material
