# Supplementary material for: Citizens’ Attitudes to Contact Tracing Apps
Source: Journal of Experimental Political Science. 2020 Sep 2:1–13. doi: 10.1017/XPS.2020.30 (PMC7653229; doi:10.1017/XPS.2020.30)
Supplement: Supplementary file 1 [file S2052263020000305sup.zip › S2052263020000305sup001.docx]

# Appendix A: Sample characteristics

Sample demographics


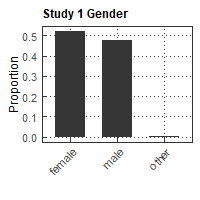

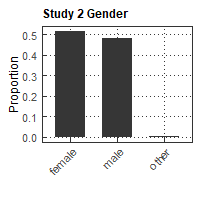


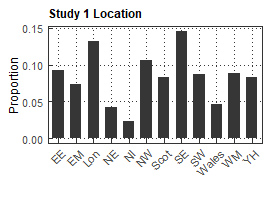

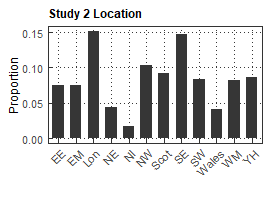


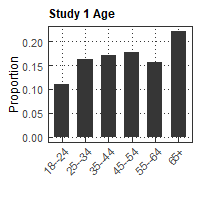

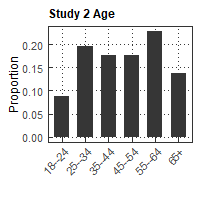


**Fig. A1**: Distribution of key sample demographics

Attention checks

Adapted from Berinsky et al. 2014:

1. In a block of four questions relating to gender discrimination, statement No. 4 read “Please click the "neither agree nor disagree" response to continue with survey.” The ratio of incorrect responses to the total number of responses is 11.77%.
2. In a block of four questions relating to attitudes to UK government policy, statement No. 4 read “Two is greater than one.” The ratio of incorrect responses to the total number of responses is 19.74%.

The distribution of conjoint preferences as well as treatment effects are robust to data quality. On the subsample of respondents who passed both checks, *N* = 1,095:


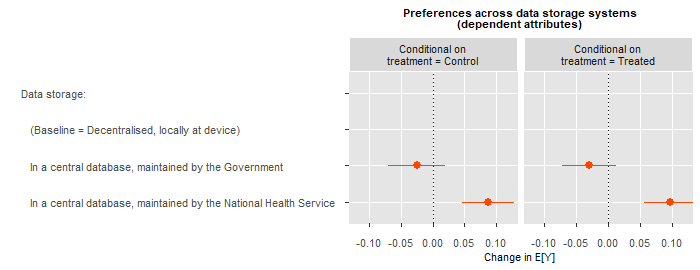


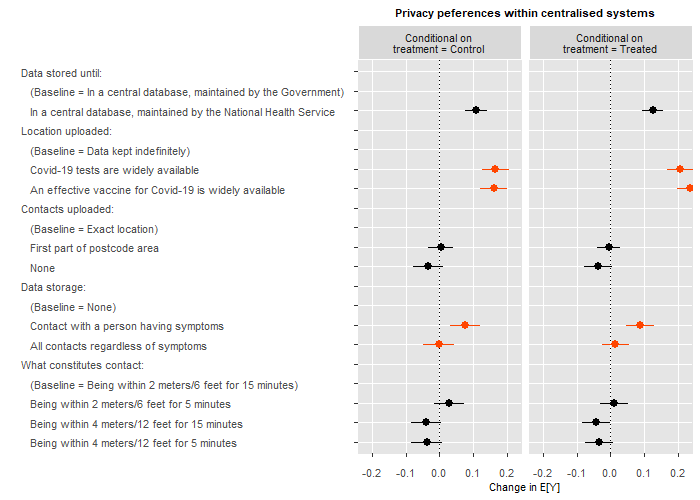


**Fig. A2**: Conjoint preferences among those who passed both attention checks

# Appendix B: Treatment details

Stimulus wording

**Table B1**: Data breach stimulus wording

| **Control group** | **Treatment** |
| --- | --- |
| Data security is a set of standards and technologies that protect data from intentional or accidental destruction, modification or disclosure.  Data security can be applied using a range of techniques and technologies, including administrative controls, physical security, and other safeguarding techniques that limit access to unauthorized or malicious users or processes. | Data security is a set of standards and technologies that protect data from intentional or accidental destruction, modification or disclosure.  Data security can be applied using a range of techniques and technologies, including administrative controls, physical security, and other safeguarding techniques that limit access to unauthorized or malicious users or processes.  Data breaches, however, are becoming more common. For example, data breaches could include hackers getting unauthorised access to or theft of personal data, devices containing personal data being lost or stolen. |

Compliance with treatment

This stimulus was not pre-tested. To scrutinise compliance with treatment, after the dependent measures, we checked respondent recall of the four terms listed (see Appendix B). Respondents in the treatment group were over two and a half times more likely to recall the term “theft of personal data” than respondents in the control group among other terms, *χ*^2^(1, 1504) = 53.4, *p* < 0.01. In Study 2, respondents in the treatment group were nearly seven times more likely to recall the this term, *χ*^2^(1, 809) = 83.1, *p* < 0.01

Randomisation result

Randomisation was successful: In Study 1 and Study 2, 52.13% and 51.66% of respondents were assigned into the data breaches group, respectively.

Display frequency of conjoint attributes

We used the Conjoint Survey Design Tool (SDT, Strezhnev et al. 2014) to program this experiment. The PHP script generating the app profiles may be viewed at the web address <http://qsteplin.ex.ac.uk/conjoint/ctrace.php>.


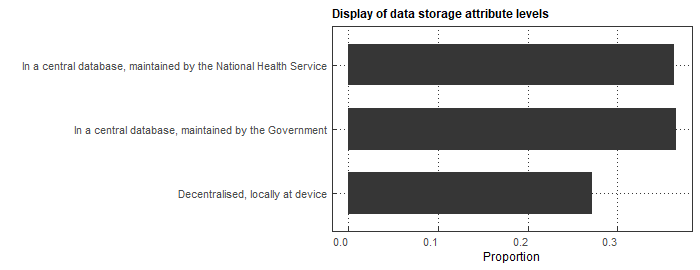


**Fig. B1**: Display frequency of data storage attribute

The various privacy attributes displayed in the app profiles depended on them being “central database” systems. We explain this reasoning behind this in Section Methods. While we note that attribute constraints are to be used sparingly in conjoint analysis (Strezhnev et al. 2014) we used this constraint to reflect the real-life choice citizens may face between a possible privacy preserving app (Troncoso et al. 2020) and a centralised system that collects protected personal data (Veale 2020). Our system-dependent attributes were resolved by the CDT program by displaying decentralised systems with somewhat lower frequency (by 9.09%). To avoid biased randomisation inference due to dependency of privacy attributes, they were only analysed on the subsample of tasks that did not involve a decentralised system---however, the results do not change significantly looking at the distribution of conjoint preferences descriptively on the full sample, see Appendix C.


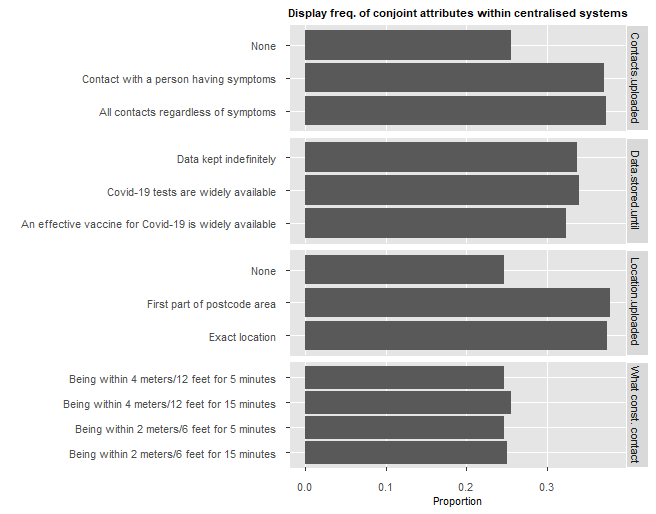


**Fig. B2**: Display frequency of privacy attributes within centralised systems

The distribution of privacy attributes is impacted by a single constraint we introduced: as centralised systems collect personal data, could not allow that “Contacts uploaded” as well as “Location uploaded” to be displayed “None” at the same time. We did however allow No Location data to be collected if some Contact data was collected and vice versa.

# Appendix C: Observed distribution of dependent measures


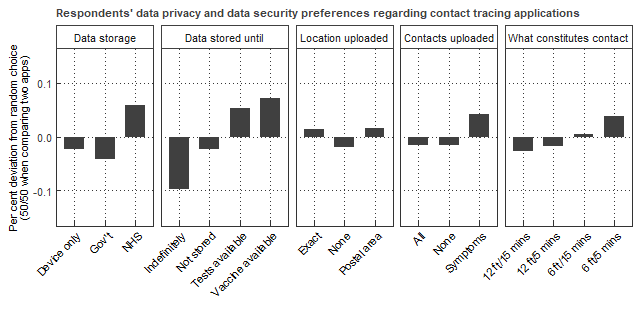


**Fig. C1**: Overview of app choice by conjoint attributes


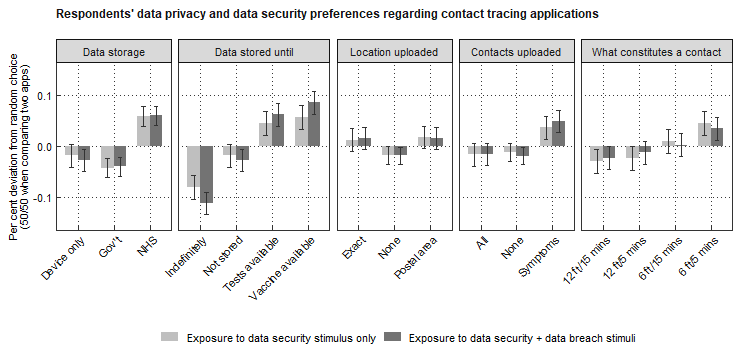


**Fig. C2**: Observed app choice by data breaches treatment group


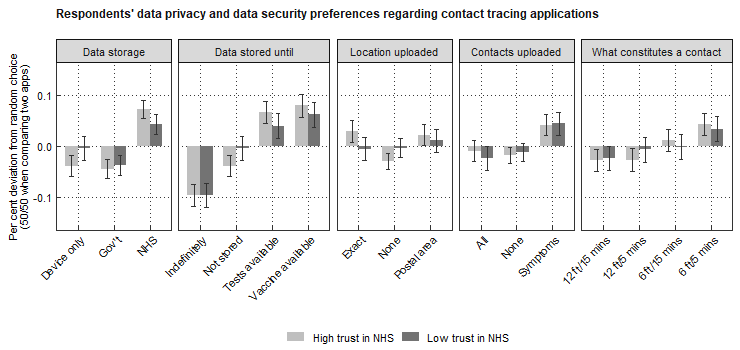


**Fig. C3**: Observed app choice by moderator 1: trust in NHS


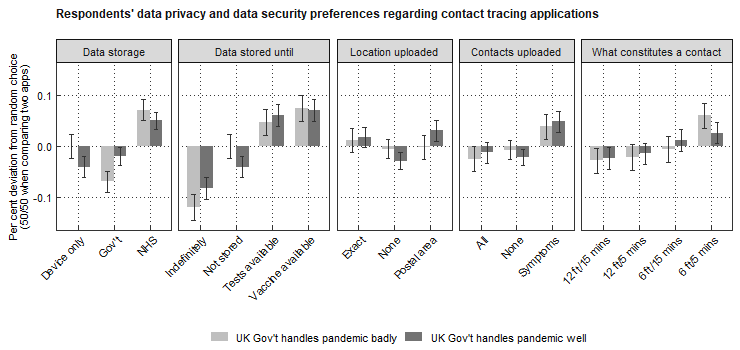


**Fig. C4**: Observed app choice by moderator 1: Government performance


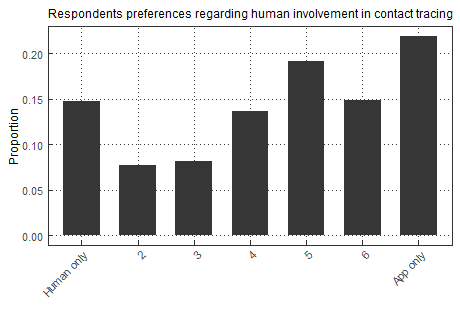


**Fig. C5**: Overview of preferences about human vs. digital contact tracing

# Appendix D: References

Berinsky, A. J., Margolis, M. F., & Sances, M. W. (2014). Separating the shirkers from the workers? Making sure respondents pay attention on self‐administered surveys. *American Journal of Political Science*, 58(3), 739-753.

Troncoso, C., Payer, M., Hubaux, J. P., Salathé, M., Larus, J., Bugnion, E., ... & Barman, L. (2020). Decentralized privacy-preserving proximity tracing. arXiv preprint: arXiv:2005.12273.

Veale, M. (2020). Analysis of the NHSX Contact Tracing App ‘Isle of Wight’ Data Protection Impact Assessment. OSF Preprint: https://osf.io/preprints/lawarxiv/6fvgh
